# A Preliminary Study on the Effects of Low Doses of Purified Zearalenone in Weaned Female Piglets: A Multi-Organ Toxicity Investigation

**DOI:** 10.3390/antiox15040496

**Published:** 2026-04-16

**Authors:** Ying Liu, Qiaomin Duan, Ruiqi Tan, Sunlin Luo, Wenjun He, Wenjun Yang, Yiqiang Chen

**Affiliations:** 1 State Key Laboratory of Animal Nutrition and Feeding, College of Animal Science and Technology, China Agricultural University, Beijing 100193, China; liuying01@cau.edu.cn (Y.L.); xiaoduo@cau.edu.cn (Q.D.); tanruiqi@cau.edu.cn (R.T.); luosunlin@cau.edu.cn (S.L.); s20233040798@cau.edu.cn (W.H.); ywj@cau.edu.cn (W.Y.); 2 Ministry of Agriculture and Rural Affairs Key Laboratory of Feed Safety and Biological Efficacy, China Agricultural University, Beijing 100193, China

**Keywords:** zearalenone, weaned piglets, oxidative stress, immunotoxicity, reproductive toxicity

## Abstract

Zearalenone (ZEA) is an estrogenic *Fusarium* mycotoxin widely contaminating feed and feedstuffs, and posing significant risks to animal health. This preliminary study aimed to evaluate the toxicological effects of dietary exposure to purified ZEA at doses ranging from below to above the Chinese regulatory limit (0.15 mg/kg) in weaned female piglets. Twenty piglets were randomly assigned to five groups (four piglets per group) receiving 0, 0.075, 0.15, 0.3, or 0.6 mg/kg ZEA for 42 days. Results suggested that ZEA promoted systemic oxidative stress, evidenced by decreased serum total antioxidant capacity (T-AOC) and increased malondialdehyde (MDA) content in liver across all doses, and in jejunal mucosa at ≥0.15 mg/kg (*p* < 0.01). Growth performance declined only at 0.6 mg/kg during days 29–42 (*p* < 0.01), while hemoglobin (HGB) levels (*p* < 0.01) and ileal villus height (*p* < 0.05) were reduced at all doses. ZEA also caused inflammatory dysregulation, as evidenced by decreased interleukin-4 (IL-4) levels in serum, liver, and intestinal tissues across all doses (*p* < 0.01), and disrupted reproductive hormones even at 0.075 mg/kg, as indicated by suppressed serum luteinizing hormone (LH) levels (*p* < 0.01), which progressed to histopathological damage in uterine and ovarian tissues at higher doses. These preliminary findings, together with significant correlations between oxidative stress markers and multi-organ parameters, suggest that low doses of purified ZEA may induce systemic oxidative stress and subclinical multi-organ toxicity in weaned female piglets, highlighting the need to incorporate redox status into risk assessment and to explore potential antioxidant-based mitigation strategies. However, given the small sample size, these results should be interpreted with caution and warrant validation in larger samples.

## 1. Introduction

Zearalenone (ZEA) is an estrogenic non-steroidal mycotoxin produced mainly by *Fusarium* fungi [1]. It widely contaminates grains such as corn, wheat, and sorghum, and can be metabolized into various derivatives, including zearalanone (ZAN), α-zearalanol (α-ZAL), β-zearalanol (β-ZAL), α-zearalenol (α-ZEL), and β-zearalenol (β-ZEL) [2,3]. Due to its environmental stability and resistance to degradation, ZEA poses a persistent threat to feed and food safety [4]. Global surveys consistently report high contamination rates of ZEA. A Brazilian study of 1749 feed and ingredient samples (2017–2021) found that 97% contained at least one mycotoxin, with ZEA detected in 62.5% at a median level of 53.3 μg/kg [5]. Similarly, a study of 400 feed samples in Spain reported ZEA in 54.3% of samples, with mean levels in positive samples ranging from 133.4 to 201.3 μg/kg across different feed types [6]. In a Kenyan survey, ZEA showed the highest contamination rate (94%) among mycotoxins in dairy and poultry feeds [7]. These findings collectively underscore the severe and pervasive contamination risk posed by ZEA in feed and feedstuffs.

Owing to its nonpolar and lipophilic properties, ZEA is readily absorbed in the animal intestine, with oral absorption rates reaching up to 85% in piglets [8]. After rapid absorption primarily in the proximal small intestine, ZEA enters systemic circulation and distributes widely to various tissues, including the liver, kidneys, muscles, and reproductive organs [9]. A portion of ZEA is excreted via feces, urine, and milk. One study reported that 76% of the administered dose (1 mg/kg) was eliminated as ZEA and its metabolites in feces and urine within 72 h in pigs [10]. ZEA is metabolized by 3α/3β-hydroxysteroid dehydrogenases to α/β-ZEL, metabolites that retain estrogenic activity with a potency order of α-ZEL > ZEA > β-ZEL [11]. This difference is attributed to their distinct binding affinities to estrogen receptors, with α-ZEL showing higher affinity and consequently exerting a more potent estrogenic effect. In pigs, α/β-ZEL can be further reduced to α/β-ZAL [12]. Among tissues, the liver typically shows the highest residual levels of ZEA and its metabolites. In farm animals, ZEA, α-ZEL, and β-ZEL are the most commonly detected residues, with ZEA and α-ZEL being the primary metabolites in pigs and poultry, whereas β-ZEL predominates in cattle tissues and milk [13].

ZEA shares a structural similarity with 17β-estradiol, which allows it to bind competitively to estrogen receptors. This interaction triggers estrogenic effects, resulting in reproductive toxicity characterized by hormonal dysregulation, organ lesions, and functional impairment [1]. In female animals, dietary ZEA exposure increases reproductive organ indices and alters tissue structure of the uterus and ovaries [14]. Chronic low-dose exposure disrupts plasma reproductive hormone levels and affects gene expression related to polycystic ovary syndrome in female rats [15]. In males, ZEA impairs the reproductive system by causing reproductive organ damage, promoting germ cell apoptosis, and lowering reproductive hormone levels [16,17]. ZEA also exhibits immunotoxic effects. In pigs, ZEA upregulates splenic pro-inflammatory cytokine gene and protein expression via c-Jun N-terminal kinase (JNK) pathway activation, disrupting immunity and inducing inflammation [18]. In mice, ZEA compromises humoral immunity by dysregulating immunoglobulins [19], while in rats, it induces thymocyte apoptosis and thymic atrophy [20]. Moreover, recent evidence indicates that ZEA disrupts intestinal barrier integrity and induces intestinal immunosuppression through alterations in intestinal flora and damage to chemical and physical barriers [21].

Critically, accumulating evidence indicates that oxidative stress serves as a pivotal mechanism underpinning ZEA-induced multi-organ toxicity. In vitro studies demonstrate that ZEA exposure disrupts cellular redox homeostasis, leading to reactive oxygen species (ROS) generation, glutathione (GSH) depletion, oxidative DNA damage, and lipid peroxidation [22,23]. Importantly, these in vitro findings are substantiated by in vivo models. Dietary ZEA has been shown to induce oxidative stress in the jejunum of postweaning gilts, as evidenced by elevated malondialdehyde (MDA) levels, decreased antioxidant enzyme activities, and activation of the Kelch-like ECH-associated protein 1-nuclear factor erythroid 2-related factor 2 (Keap1-Nrf2) signaling pathway [24]. More recently, ZEA has been demonstrated to induce oxidative stress and apoptosis in the jejunum of weaned piglets through the p53/Nrf2 signaling pathway [25]. Similarly, ZEA exposure promotes hepatic oxidative stress in piglets, which can be effectively mitigated by the antioxidant vitamin C, highlighting the central role of redox imbalance in ZEA-induced liver damage [26].

The established toxicity of zearalenone has led to the adoption of feed safety regulations in major agricultural countries, where maximum permissible levels for ZEA are enforced to control exposure risks. In China, the *Hygienical Standard for Feeds* (GB 13078-2017) sets the maximum ZEA level in piglet feed at 0.15 mg/kg [27], while the European Union imposes a stricter limit of 0.1 mg/kg [28]. Although many toxicological studies use doses far exceeding regulatory thresholds, actual exposure concentrations in feed generally remain relatively low [4,29]. Moreover, in studies using naturally contaminated diets, observed effects may be confounded by ZEA derivatives, other co-occurring contaminants, or reduced nutritional quality of mold-affected feed. We hypothesized that dietary exposure to purified ZEA at low doses would induce systemic oxidative stress, thereby leading to subclinical multi-organ toxicity. To test this hypothesis, we conducted a preliminary study including a systematic evaluation of its impact on growth performance, blood biochemistry, antioxidant function, immune responses, intestinal health, and reproductive development in weaned female piglets. In addition, Spearman correlation analysis was performed to further explore the associations between oxidative stress markers and multi-organ parameters, aiming to provide statistical evidence linking redox imbalance to the observed toxicities. These results are expected to provide a scientific basis for optimizing mycotoxin control strategies in feed, with a particular focus on redox status as a sensitive endpoint for risk assessment, and to inform potential revisions to regulatory limits.

## 2. Materials and Methods

### 2.1. Standards, Animals, and Ethics

The ZEA standard (>98% purity) used in this experiment was purchased from Shandong Meizheng Bio-tech Co., Ltd. (Rizhao, China). A total of twenty female Duroc × Landrace × Yorkshire piglets (28 days old, average body weight 7.12 ± 0.40 kg) were obtained from the Fengning Research Unit of China Agricultural University. Only female piglets were used in this study because they are more sensitive to the estrogenic effects of ZEA, allowing for a clearer assessment of reproductive toxicity. Approval for all procedures in this study was granted by the China Agricultural University Laboratory Animal Welfare and Animal Experimental Ethical Inspection Committee (Approval No. AW40704202–1–4, 4 July 2024). All procedures were conducted in strict accordance with relevant ethical guidelines.

### 2.2. Experimental Design, Diets, and Management

A total of twenty female weaned piglets were randomly allocated to five dietary treatments, with four replicates per treatment and one pig per replicate. The piglets were individually housed in stainless-steel cages (dimensions: 1.4 × 0.7 × 0.6 m) for a 42-day trial period. A corn-soybean meal basal diet, formulated to meet NRC (2012) nutrient requirements of piglets [30], was used throughout the experiment. The treatment groups were designated as CON, ZEA0.075, ZEA0.15, ZEA0.3, and ZEA0.6, corresponding to the supplementation of purified ZEA at concentrations of 0, 0.075, 0.15, 0.3, and 0.6 mg/kg, respectively, into the basal diet. To prepare the ZEA-contaminated diets, a pre-weighed amount of ZEA powder was first dissolved in 10 mL of anhydrous ethanol. This solution was then thoroughly premixed with approximately 5 kg of the basal diet. The premix was subsequently blended with the remaining quantity of the basal diet using a multi-function feed mixer to achieve the target final concentrations. The control diet (CON group) was prepared by adding an equal volume of anhydrous ethanol (10 mL) to the basal diet, followed by the same mixing procedure as the treatment diets to serve as a true vehicle control. The formulation and nutritional composition of the basal diet were identical to those reported in our previous study [31]. The concentrations of ZEA and five other major mycotoxins (deoxynivalenol, DON; aflatoxin B_1_, AFB_1_; ochratoxin A, OTA; T-2 toxin; and fumonisin B_1_, FB_1_) in the experimental diets were determined using our published method [32], with the results detailed in Supplementary Table S1. Prior to the experiment, the pen was thoroughly disinfected, and cages along with feeders were cleaned. Ambient temperature was maintained at 24–26 °C with adequate ventilation. Throughout the study, piglets had ad libitum access to feed and water. Routine disinfection, vaccination, and deworming protocols were followed.

### 2.3. Blood Collection and Tissue Harvesting

Blood sampling was performed on days 14, 28, and 42 after a 12 h fasting period. All piglets were bled via the anterior vena cava. For hematology, 3 mL of whole blood was immediately placed into anticoagulant tubes and kept at 4 °C. For serum separation, a further 10 mL was drawn into plain tubes, allowed to clot for 30 min at room temperature, and centrifuged (3000× *g*, 10 min). The supernatant serum was aliquoted and stored at −20 °C for subsequent biochemical assays.

On the final experimental day (day 42), all piglets were euthanized by intravenous administration of an overdose of sodium pentobarbital. For organ index assessment, the heart, liver, spleen, lungs, and kidneys were rapidly dissected, cleared of connective tissue, and weighed. For histopathology, tissue specimens from the liver, kidneys, duodenum, jejunum, ileum, uterus, and ovaries were harvested. These specimens were briefly rinsed in 0.9% physiological saline to remove residual blood, then immersion-fixed in 4% paraformaldehyde buffer. Fixed intestinal segments were further used for later morphological evaluation. Additionally, for tissue biochemical analyses, liver tissue samples were collected, and mucosal scrapings from the jejunum and ileum were obtained using glass slides. All such samples for biochemical assays were rapidly flash-frozen in liquid nitrogen and maintained at −80 °C until further analysis.

### 2.4. Growth Performance and Organ Indices

The initial body weight (IBW) of each piglet was recorded at the start of the trial. Fasting body weights were subsequently measured on days 14, 28, and 42, and feed consumption was recorded throughout the experimental period. These data were used to calculate average daily gain (ADG), average daily feed intake (ADFI), and the feed-to-gain ratio (F: G) for the periods of 0–14, 15–28, and 29–42 days, as well as for the overall 0–42 day period. Organ indices for the heart, liver, spleen, lungs, and kidneys were determined using the following formula: organ index (g/kg) = organ weight/final body weight.

### 2.5. Hematological and Serum Biochemical Analyses

Hematological parameters were assessed using a fully automated hematology analyzer (XS-800i, Sysmex Corporation, Kobe, Japan). The measured indices included white blood cell count (WBC), red blood cell count (RBC), hemoglobin (HGB), hematocrit (HCT), mean corpuscular volume (MCV), mean corpuscular hemoglobin (MCH), mean corpuscular hemoglobin concentration (MCHC), standard deviation of red cell distribution width (RDW-SD), coefficient of variation in red cell distribution width (RDW-CV), platelet count (PLT), platelet distribution width (PDW), and mean platelet volume (MPV). Serum biochemical analyses were performed on a fully automated biochemistry analyzer (BS-420, Shenzhen Mindray Bio-Medical Electronics Co., Ltd., Shenzhen, China) with corresponding commercial assay kits (Zhongsheng Beikong Bio-technology Co., Ltd., Beijing, China). The levels of total protein (TP), albumin (ALB), globulin (GLB), creatinine (CREA), urea nitrogen (UN), glucose (GLU), aspartate aminotransferase (AST), alanine aminotransferase (ALT), total bilirubin (TBIL), alkaline phosphatase (ALP), cholinesterase (CHE), and lactate dehydrogenase (LDH) were quantified.

### 2.6. Assessment of Antioxidant Function

A panel of redox status biomarkers was evaluated to assess systemic and tissue-specific antioxidant function. Specifically, the activities of glutathione peroxidase (GSH-Px) and superoxide dismutase (SOD), the total antioxidant capacity (T-AOC), and the concentration of malondialdehyde (MDA) were determined in serum, liver, and the mucosa of the jejunum and ileum. All assays were conducted strictly following the manufacturer’s protocols using commercial colorimetric kits (Beijing Sinouk Institute of Biological Technology, Beijing, China). Absorbance was read on a microplate reader (DR-200BS, Wuxi Hiwell-Diatek Instruments Co., Ltd., Wuxi, China).

### 2.7. Measurement of Immune Parameters

The immune response of piglets exposed to ZEA was assessed by measuring immunoglobulin levels and cytokine concentrations in serum and tissue samples. Specifically, immunoglobulin A (IgA), immunoglobulin G (IgG), and immunoglobulin M (IgM) levels in serum and liver were determined using colorimetric methods on the fully automated biochemical analyzer described in Section 2.5. The levels of tumor necrosis factor-α (TNF-α), interleukin-1β (IL-1β), interleukin-2 (IL-2), interleukin-4 (IL-4), and interleukin-10 (IL-10) in serum, liver, and the mucosa of the jejunum and ileum were measured using enzyme-linked immunosorbent assay (ELISA) kits (Beijing Sinouk Institute of Biological Technology, Beijing, China), with absorbance readings performed on the microplate reader described in Section 2.6. All assays were conducted in strict accordance with the manufacturers’ protocols.

### 2.8. Evaluation of Intestinal Morphology and Histopathology

Fixed tissues (including the liver, kidneys, duodenum, jejunum, ileum, uterus, and ovaries) were trimmed and subjected to dehydration, clearing, paraffin embedding, sectioning, and hematoxylin-eosin (HE) staining. The resulting HE-stained sections were observed under an upright microscope (Primo Star, Carl Zeiss, Oberkochen, Germany). Morphometric analysis of the intestine was conducted by capturing images with Image-Pro Plus 6.0 software. Villus height (VH) and crypt depth (CD) were measured on 10 intact villi per sample, and their mean values served to calculate the villus height-to-crypt depth ratio (VH/CD). A comprehensive histopathological examination was performed on sections from all organs to assess tissue integrity and detect pathological alterations.

### 2.9. Determination of Intestinal Barrier Integrity

Intestinal barrier integrity was evaluated by measuring serum levels of lipopolysaccharide (LPS), diamine oxidase (DAO), and D-lactic acid (D-LA) using commercial colorimetric assay kits (Beijing Sinouk Institute of Biological Technology, Beijing, China). All procedures were conducted following the manufacturer’s instructions, with the same microplate reader described in Section 2.6.

### 2.10. Analysis of Reproductive Hormone Profiles

Commercial ELISA kits (Beijing Sinouk Institute of Biological Technology, Beijing, China) were employed to determine serum concentrations of follicle-stimulating hormone (FSH), luteinizing hormone (LH), estradiol (E_2_), gonadotropin-releasing hormone (GnRH), prolactin (PRL), and progesterone (PROG). The assay procedures were strictly followed according to the manufacturer’s protocols, utilizing the microplate reader described in Section 2.6.

### 2.11. Data Analysis

The trial was conducted as a randomized complete block design, with piglets blocked by initial body weight and randomly assigned to five treatments. Each piglet was housed individually and served as the experimental unit for all measured parameters. The trial data were analyzed by one-way analysis of variance (ANOVA) using the General Linear Model (GLM) procedure in SAS 9.4 software [33], followed by Duncan’s multiple range test for multiple comparisons. Differences were considered statistically significant at *p* < 0.05 and highly significant at *p* < 0.01. Spearman correlation analysis was applied to further explore potential associations between oxidative stress markers and multi-organ parameters. Given the small sample size (*n* = 4 per group), the results should be interpreted with caution. Dose-dependent trend analyses (linear and quadratic) are recommended for future studies with larger sample sizes to better characterize dose–response relationships.

## 3. Results

### 3.1. Effects of Purified ZEA on Growth Performance of Piglets

As presented in Table 1, there were no significant differences (*p* > 0.05) in growth performance among all treatment groups during the phases of days 0–14, 15–28, and 0–42. However, during days 29–42, the ZEA0.6 group showed a significant increase in the feed-to-gain ratio (F:G) compared with the CON group (from 1.87 in the CON group to 2.06 in the ZEA0.6 group, *p* < 0.01).

### 3.2. Effects of Purified ZEA on Major Organ Indices of Piglets

The major organ indices of piglets were not affected by dietary ZEA exposure, as detailed in Table 2. Specifically, no significant differences were observed in the indices of the heart, liver, spleen, lungs or kidneys among all treatment groups (*p* > 0.05).

### 3.3. Effects of Purified ZEA on Hematological and Biochemical Parameters of Piglets

Dietary ZEA exposure induced perturbations in some hematological and biochemical parameters in piglets. Hematological results for day 42 are presented in Table 3, and the corresponding data for days 14 and 28 are provided in Supplementary Table S2. No significant changes in any hematological parameters were observed on days 14 or 28. On day 42, however, hemoglobin (HGB) levels were significantly decreased in all ZEA-treated groups (*p* < 0.01). Furthermore, a reduction in hematocrit (HCT) was noted at the ZEA0.15, ZEA0.3, and ZEA0.6 groups (*p* < 0.05). Specifically, the ZEA0.075 group exhibited decreases in platelet distribution width (PDW) and mean platelet volume (MPV) (*p* < 0.01).

Similarly, serum biochemistry results for day 42 are shown in Table 4, while those for days 14 and 28 are detailed in Supplementary Table S3. On day 14, total protein (TP) was significantly elevated in the ZEA0.3 group (*p* < 0.05). Aspartate aminotransferase (AST) activity increased in the ZEA0.075 and ZEA0.6 groups on day 28 (*p* < 0.05), and this increase persisted in the ZEA0.075 group on day 42 (*p* < 0.05).

### 3.4. Effects of Purified ZEA on Antioxidant Function of Piglets

Serum antioxidant results are presented in Table 5. On day 14, the ZEA0.075 group showed a significant reduction in serum glutathione peroxidase (GSH-Px) activity (*p* < 0.01), and all ZEA-treated groups had lower total antioxidant capacity (T-AOC) (*p* < 0.01). On day 28, superoxide dismutase (SOD) activity was decreased in the ZEA0.15, ZEA0.3, and ZEA0.6 groups (*p* < 0.01), while T-AOC remained reduced across all ZEA groups (*p* < 0.01). By day 42, serum GSH-Px activity was suppressed in all ZEA-treated groups (*p* < 0.01).

**Figure 1:**
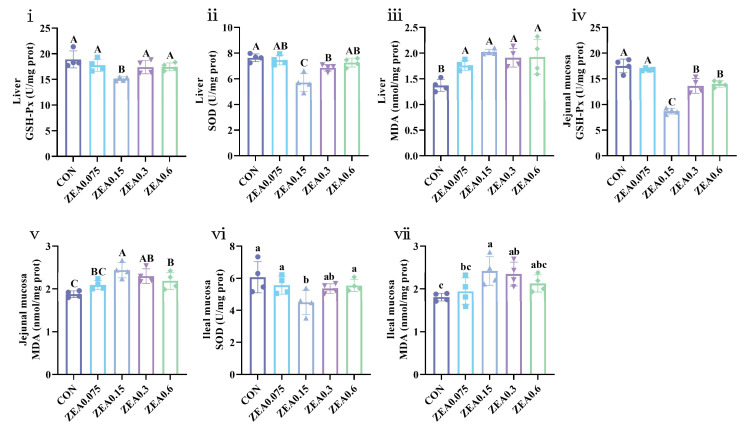
Effects of ZEA exposure on antioxidant parameters in the liver and intestine of piglets. (**i**) Glutathione peroxidase (GSH-Px) activity, (**ii**) superoxide dismutase (SOD) activity and (**iii**) malondialdehyde (MDA) content in the liver. (**iv**) GSH-Px activity and (**v**) MDA content in the jejunal mucosa. (**vi**) SOD activity and (**vii**) MDA content in the ileal mucosa. Data are presented as mean ± SD, *n* = 4. Four symbols with the same color and shape per group represent the individual values of the four replicates. A–C, different letters mean a statistical difference (*p* < 0.01). a–c, different letters mean a statistical difference (*p* < 0.05).

Data on liver and intestinal antioxidant parameters are provided in Supplementary Table S4, with statistically significant changes summarized in Figure 1. In liver tissue, GSH-Px activity was lower in the ZEA0.15 group (*p* < 0.01). Liver SOD activity was reduced in the ZEA0.15 and ZEA0.3 groups (*p* < 0.01), while MDA content was elevated in all ZEA-treated groups (*p* < 0.01). In the jejunal mucosa, GSH-Px activity was decreased and MDA content was increased in the ZEA0.15, ZEA0.3, and ZEA0.6 groups (*p* < 0.01). In the ileal mucosa, SOD activity was lower in the ZEA0.15 group (*p* < 0.05), and MDA content was higher in the ZEA0.15 and ZEA0.3 groups (*p* < 0.05).

### 3.5. Effects of Purified ZEA on Immune Parameters of Piglets

Serum immunoglobulin results are summarized in Table 6. Compared with the CON group, the ZEA0.3 group showed elevated serum levels of immunoglobulin A (IgA), immunoglobulin G (IgG), and immunoglobulin M (IgM) on day 14 (*p* < 0.01). On day 28, serum IgM levels were higher in the ZEA0.6 group (*p* < 0.01). However, by day 42, no significant differences in serum IgA, IgG, or IgM levels were observed across all ZEA-treated groups (*p* > 0.05). Similarly, levels of IgA, IgG, and IgM in the liver did not differ among all groups (*p* > 0.05; Supplementary Figure S1).

**Figure 2:**
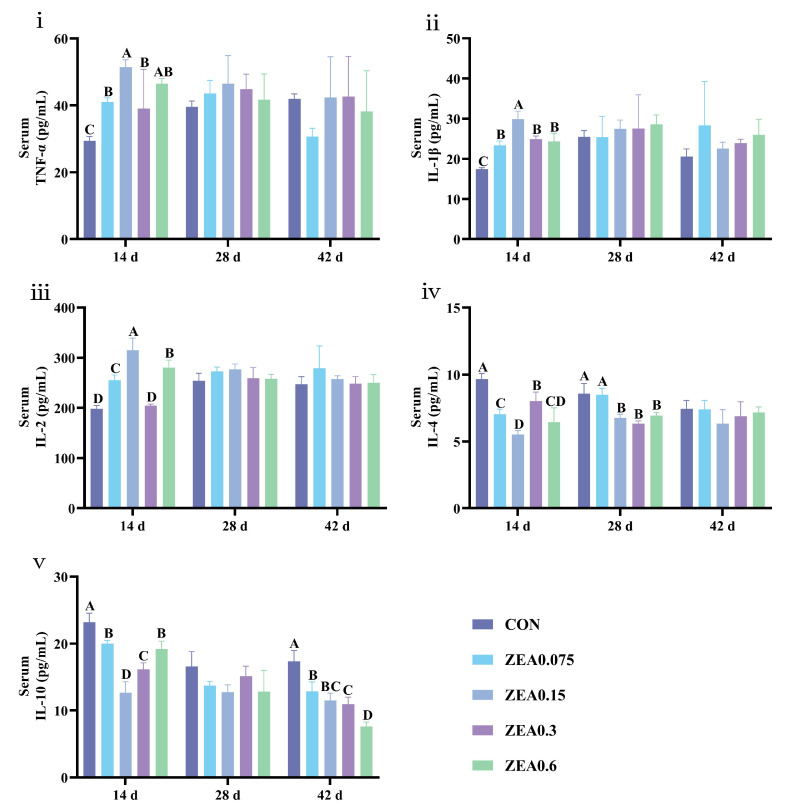
Effects of ZEA exposure on serum immune cytokine levels of piglets. (**i**) Tumor necrosis factor-α (TNF-α), (**ii**) Interleukin-1β (IL-1β), (**iii**) Interleukin-2 (IL-2), (**iv**) Interleukin-4 (IL-4), and (**v**) Interleukin-10 (IL-10). Data are presented as mean ± SD, *n* = 4. A–D, different letters mean a statistical difference (*p* < 0.01).

Serum immune cytokine results are shown in Figure 2. On day 14, all ZEA groups exhibited increased tumor necrosis factor-α (TNF-α) and interleukin-1β (IL-1β) levels alongside decreased interleukin-4 (IL-4) and interleukin-10 (IL-10) levels (*p* < 0.01). Interleukin-2 (IL-2) levels were elevated in the ZEA0.075, ZEA0.15, and ZEA0.6 groups (*p* < 0.01). On day 28, IL-4 levels were lower in the ZEA0.15, ZEA0.3, and ZEA0.6 groups (*p* < 0.01). On day 42, dietary ZEA exposure significantly reduced IL-10 levels (*p* < 0.01), while TNF-α, IL-1β, IL-2, and IL-4 remained unaffected (*p* > 0.05).

**Figure 3:**
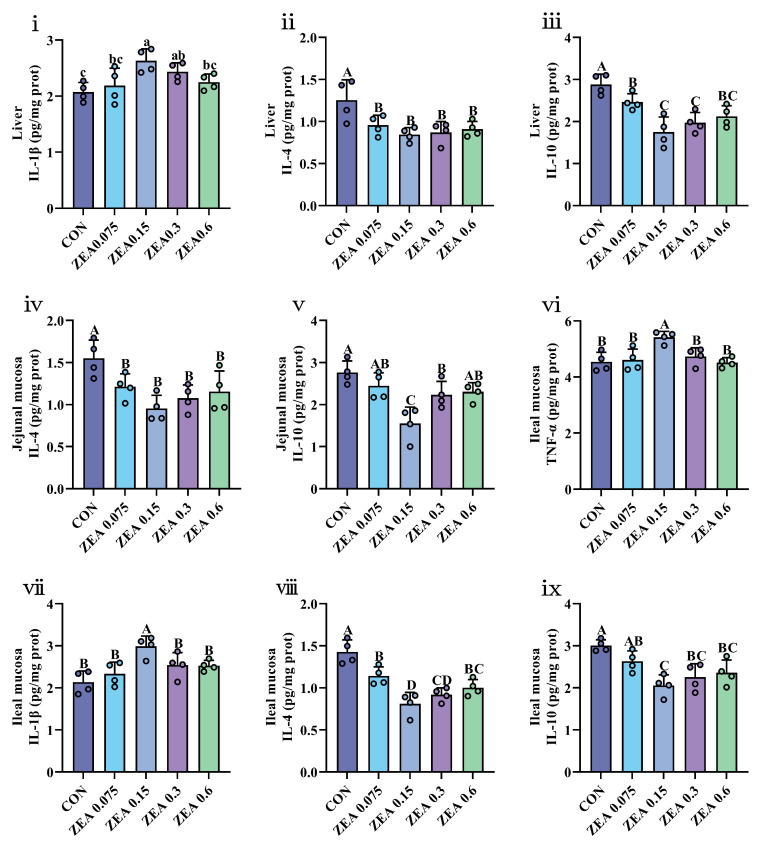
Effects of ZEA exposure on immune cytokine levels in the liver and intestine of piglets. (**i**) Interleukin-1β (IL-1β), (**ii**) Interleukin-4 (IL-4), and (**iii**) Interleukin-10 (IL-10) levels in the liver. (**iv**) IL-4 and (**v**) IL-10 levels in the jejunal mucosa. (**vi**) Tumor necrosis factor-α (TNF-α), (**vii**) IL-1β, (**viii**) IL-4 and (**ix**) IL-10 levels in the ileal mucosa. Data are presented as mean ± SD, *n* = 4. Four circles with the same color per group represent the individual values of the four replicates. A–D, different letters mean a statistical difference (*p* < 0.01). a–c, different letters mean a statistical difference (*p* < 0.05).

Hepatic and intestinal immune cytokine profiles are detailed in Supplementary Table S5, with significant findings illustrated in Figure 3. In the liver, IL-1β levels were increased in the ZEA0.15 and ZEA0.3 groups (*p* < 0.05), while IL-4 and IL-10 levels were decreased across all ZEA-treated groups (*p* < 0.01). In the jejunal mucosa, IL-4 was lower in all ZEA-treated groups (*p* < 0.01), and IL-10 was reduced in the ZEA0.15 and ZEA0.3 groups (*p* < 0.01). In the ileal mucosa, TNF-α and IL-1β were elevated in the ZEA0.15 group (*p* < 0.01). IL-4 was decreased in all ZEA groups (*p* < 0.01), and IL-10 was lower in the ZEA0.15, ZEA0.3, and ZEA0.6 groups (*p* < 0.01).

### 3.6. Effects of Purified ZEA on Intestinal Morphology and Histopathology of Piglets

**Figure 4:**
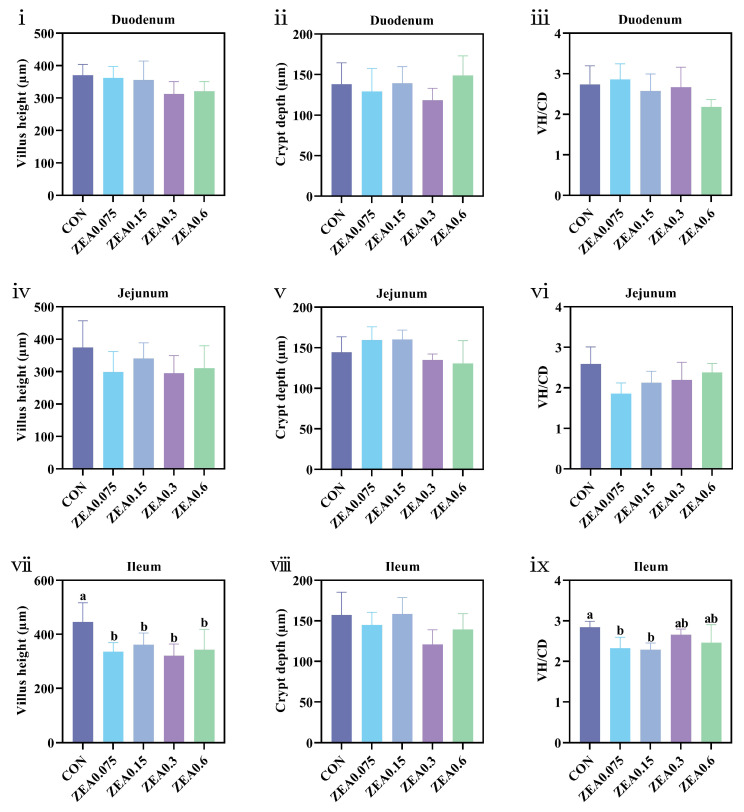
Intestinal morphology of piglets exposed to dietary purified ZEA. (**i**–**iii**) Duodenal villus height (VH), crypt depth (CD), and VH/CD ratio. (**iv**–**vi**) Jejunal VH, CD, and VH/CD ratio. (**vii**–**ix**) Ileal VH, CD, and VH/CD ratio. Data are presented as mean ± SD, *n* = 4. a, b, different letters mean a statistical difference (*p* < 0.05).

Figure 4 presents the quantitative analysis of intestinal morphology. While duodenal and jejunal morphology remained unaffected by dietary purified ZEA exposure (*p* > 0.05), ileal alterations were evident. All ZEA-treated groups showed a decrease in ileal villus height (VH) (*p* < 0.05), and the ZEA0.075 and ZEA0.15 groups had a reduced VH-to-crypt depth (VH/CD) ratio (*p* < 0.05).

**Figure 5:**
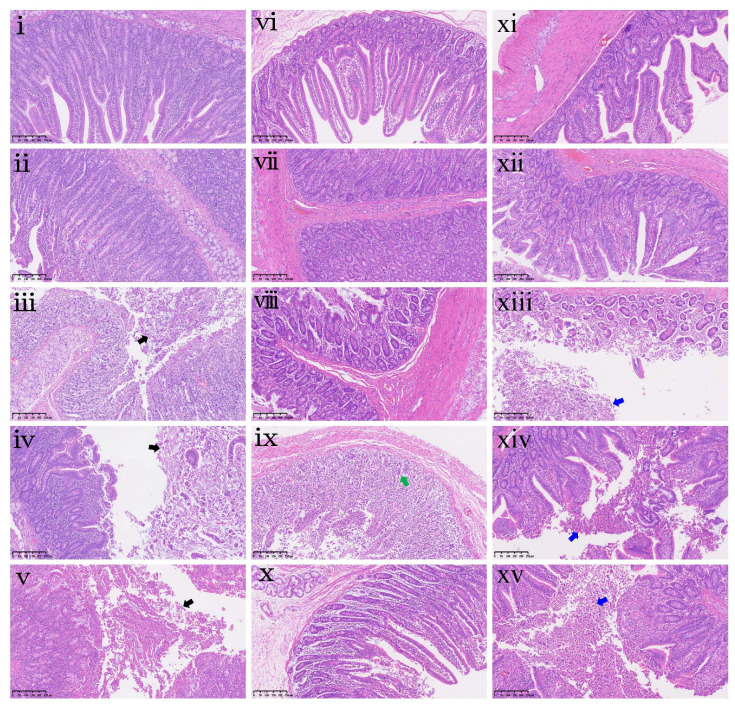
Intestinal histopathological examination of piglets exposed to dietary purified ZEA. (**i**–**v**) Representative duodenum sections, (**vi**–**x**) jejunum sections, and (**xi**–**xv**) ileum sections from piglets in the CON, ZEA0.075, ZEA0.15, ZEA0.3 and ZEA0.6 groups, respectively. Pathological findings are marked with colored arrows. Black arrows indicate mild sloughing of mucosal components, green arrows indicate moderate mononuclear cell hyperplasia, and blue arrows indicate mild to moderate epithelial detachment and accumulation. All specimens were examined at 100× magnification. Scale bar = 250 μm.

Histopathological assessment (Figure 5) was conducted to complement these morphometric findings, revealing lesions that extended beyond the quantitative measurements. In the duodenum, ZEA0.15, ZEA0.3, and ZEA0.6 groups exhibited mild sloughing of mucosal components (including epithelium, stromal cells, and goblet cells) into the lumen (Figure 5iii–v). In the jejunum, moderate mononuclear cell hyperplasia in the lamina propria was noted in the ZEA0.3 group (Figure 5ix). In the ileum, epithelial detachment and accumulation, ranging from mild (Figure 5xiii,xiv) to moderate (Figure 5xv), were observed in the ZEA0.15, ZEA0.3 and ZEA0.6 groups.

### 3.7. Effects of Purified ZEA on Intestinal Barrier Function of Piglets

**Figure 6:**
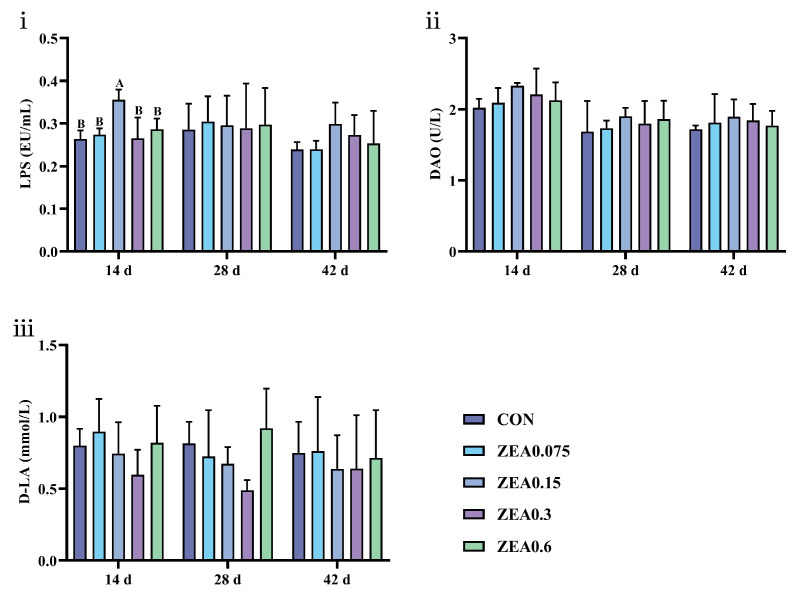
Effects of ZEA exposure on intestinal barrier function of piglets. (**i**) Serum lipopolysaccharide (LPS), (**ii**) diamine oxidase (DAO), and (**iii**) D-lactic acid (D-LA) levels. Data are presented as mean ± SD, *n* = 4. A, B, different letters mean a statistical difference (*p* < 0.01).

Analysis of intestinal barrier function is presented in Figure 6. Dietary purified ZEA exposure showed a limited and early-phase effect. On day 14, serum lipopolysaccharide (LPS) levels were elevated in the ZEA0.15 group (*p* < 0.01). However, this effect was not sustained. On days 28 and 42, no significant differences were observed in serum levels of LPS, diamine oxidase (DAO), or D-lactic acid (D-LA) among all ZEA-treated groups (*p* > 0.05). These findings suggest that purified ZEA, at the tested doses, did not induce persistent increases in serum markers of intestinal permeability, despite the evident structural damage observed in the intestinal mucosa.

### 3.8. Effects of Purified ZEA on Hepatic and Renal Histopathology of Piglets

**Figure 7:**
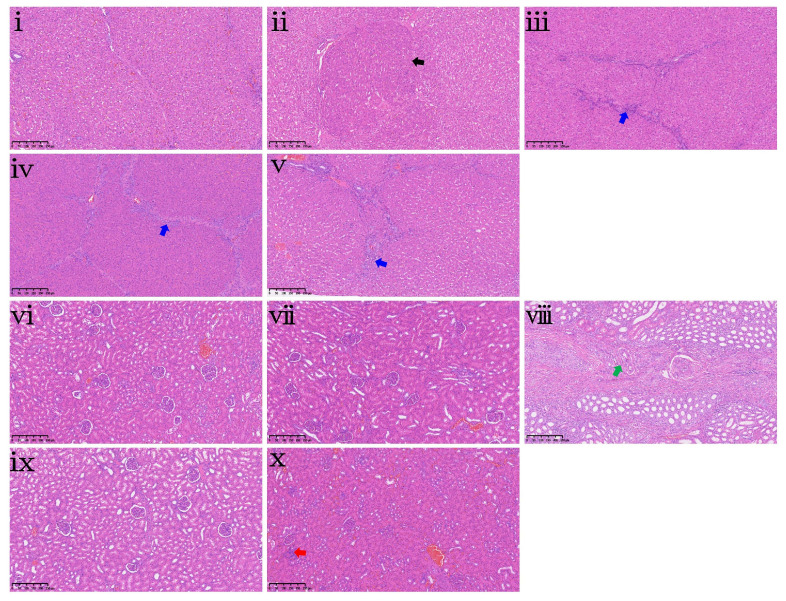
Hepatic and renal histopathological examination of piglets exposed to dietary purified ZEA. (**i**–**v**) Representative liver sections and (**vi**–**x**) kidney sections from piglets in the CON, ZEA0.075, ZEA0.15, ZEA0.3 and ZEA0.6 groups, respectively. Pathological findings are marked with colored arrows. Black arrow indicates mild multifocal non-regenerative hyperplasia, blue arrows indicate mild biliary duct hyperplasia, the green arrow indicates mild perivascular fibrosis, and the red arrow indicates mild interstitial inflammatory cell infiltration. All specimens were examined at 100× magnification. Scale bar = 250 μm.

Histopathological analysis of the liver and kidney is shown in Figure 7. In the liver, mild multifocal non-regenerative hyperplasia was observed in the ZEA0.075 group (Figure 7ii), and mild biliary duct hyperplasia was noted in the ZEA0.15, ZEA0.3, and ZEA0.6 groups (Figure 7iii–v). In the kidney, mild perivascular fibrosis was present in the ZEA0.15 group (Figure 7viii), while mild interstitial inflammatory cell infiltration was found in the ZEA0.6 group (Figure 7x).

### 3.9. Effects of Purified ZEA on Reproductive Hormone Profiles and Reproductive Organ Histopathology of Piglets

**Figure 8:**
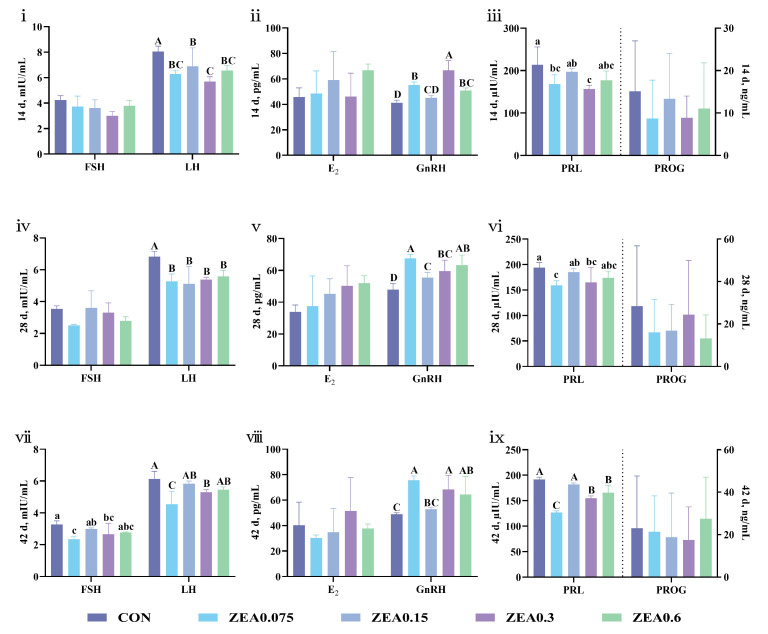
Serum reproductive hormone changes in piglets exposed to dietary purified ZEA. Serum levels of follicle-stimulating hormone (FSH), luteinizing hormone (LH), estradiol (E_2_), gonadotropin-releasing hormone (GnRH), prolactin (PRL), and progesterone (PROG) were measured on (**i**–**iii**) day 14, (**iv**–**vi**) day 28, and (**vii**–**ix**) day 42. Data are presented as mean ± SD, *n* = 4. A–D, different letters mean a statistical difference (*p* < 0.01). a–c, different letters mean a statistical difference (*p* < 0.05).

Alterations in reproductive hormone profiles following purified ZEA exposure are summarized in Figure 8. On day 14, serum luteinizing hormone (LH) levels were decreased in all ZEA-treated groups (*p* < 0.01). Serum gonadotropin-releasing hormone (GnRH) levels were elevated in the ZEA0.075, ZEA0.3, and ZEA0.6 groups (*p* < 0.01), while prolactin (PRL) levels were reduced in the ZEA0.075 and ZEA0.3 groups (*p* < 0.05). On day 28, a sustained suppression of LH was observed across all ZEA groups (*p* < 0.01), accompanied by increased GnRH levels (*p* < 0.01). Serum PRL levels remained lower in the ZEA0.075 and ZEA0.3 groups (*p* < 0.05). By day 42, the hormonal disruptions persisted and expanded. Serum follicle-stimulating hormone (FSH) and LH levels were reduced in the ZEA0.075 and ZEA0.3 groups (*p* < 0.05 and *p* < 0.01, respectively). Concurrently, GnRH levels were elevated in the ZEA0.075, ZEA0.3, and ZEA0.6 groups (*p* < 0.01), and PRL levels were decreased in these groups (*p* < 0.01).

**Figure 9:**
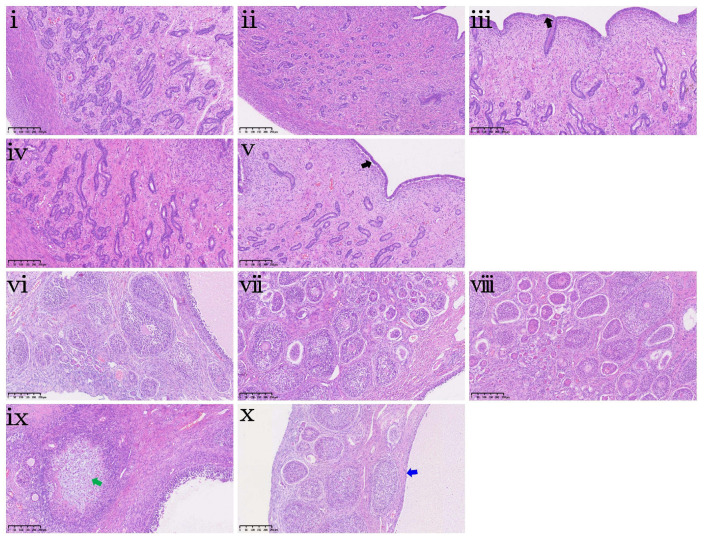
Histopathological changes in the uterus and ovary of piglets exposed to dietary purified ZEA. (**i**–**v**) Representative uterus sections and (**vi**–**x**) ovary sections from piglets in the CON, ZEA0.075, ZEA0.15, ZEA0.3 and ZEA0.6 groups, respectively. Pathological findings are marked with colored arrows. The black arrow indicates mild subepithelial cellular vacuolization in the endometrium, the green arrow indicates mild intrafollicular mucus accumulation, and the blue arrow indicates follicular cysts. All specimens were examined at 100× magnification. Scale bar = 250 μm.

Further histopathological examination of the uterus and ovary (Figure 9) revealed mild, tissue-specific pathological damage. In the uterus, mild subepithelial cellular vacuolization in the endometrium was noted in the ZEA0.15 and ZEA0.6 groups (Figure 9iii,v). In the ovary, mild intrafollicular mucus accumulation was observed in the ZEA0.3 group (Figure 9ix), while follicular cysts were present in the ZEA0.6 group (Figure 9x).

### 3.10. Correlation Analysis Between Oxidative Stress Markers and Multi-Organ Parameters

**Figure 10:**
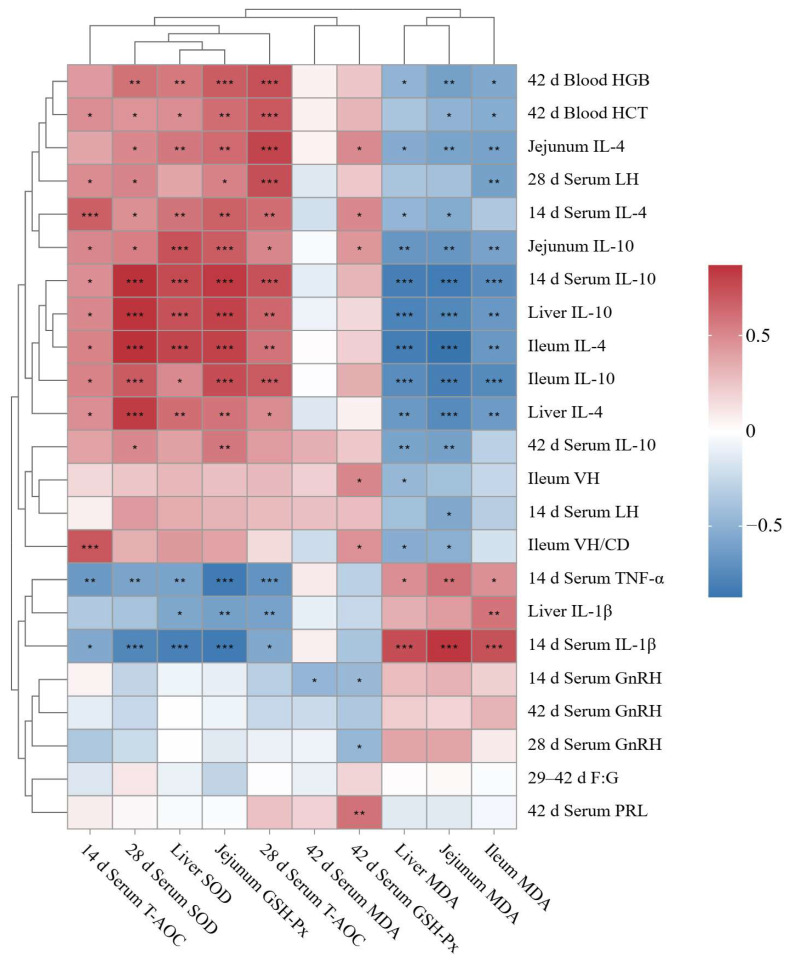
Spearman correlation heatmap between oxidative stress markers and multi-organ parameters in piglets. T-AOC, total antioxidant capacity; SOD, superoxide dismutase; GSH-Px, glutathione peroxidase; MDA, malondialdehyde; F:G, feed to gain ratio; HGB, hemoglobin; HCT, hematocrit; TNF-α, tumor necrosis factor-α; IL-1β, interleukin-1β; IL-4, interleukin-4; IL-10, interleukin-10; VH, villus height; VH/CD, villus height to crypt depth ratio; LH, luteinizing hormone; GnRH, gonadotropin-releasing hormone; PRL, prolactin. * *p* < 0.05, ** *p* < 0.01, *** *p* < 0.001.

To further investigate the associations between ZEA-induced oxidative stress and multi-organ alterations, Spearman correlation analysis was performed. As shown in Figure 10, antioxidant parameters in serum and tissues, including serum T-AOC on day 14, serum SOD on day 28, serum T-AOC on day 28, liver SOD, and jejunal GSH-Px, were significantly positively correlated with anti-inflammatory cytokines (IL-4 and IL-10) in the serum, liver, jejunum, and ileum, as well as with blood HGB and HCT on day 42 and serum LH on day 28 (*p* < 0.05). In contrast, these antioxidant markers were negatively correlated with the pro-inflammatory cytokines serum TNF-α and IL-1β on day 14 (*p* < 0.05). Conversely, MDA content in the liver, jejunum, and ileum was significantly positively correlated with serum TNF-α and IL-1β on day 14 (*p* < 0.05), and negatively correlated with IL-4 and IL-10 in the liver, jejunum, and ileum, as well as with serum IL-4 and IL-10 on day 14, and with blood HGB on day 42 (*p* < 0.05). In addition, serum GSH-Px on day 42 was positively correlated with ileal VH and the VH/CD ratio, whereas liver MDA content was negatively correlated with these parameters (*p* < 0.05). Moreover, serum GSH-Px on day 42 was positively correlated with serum PRL on day 42, but negatively correlated with serum GnRH on day 14 and day 28 (*p* < 0.05), whereas jejunal MDA content was negatively correlated with serum LH on day 14, and ileal MDA content with serum LH on day 28 (*p* < 0.05).

## 4. Discussion

In this preliminary study, disruption of the redox balance and induction of oxidative stress may represent a major pathway through which ZEA exerts its toxic effects. A pivotal study demonstrated that dietary inclusion of 1 mg/kg purified ZEA markedly increased hepatic MDA levels while decreasing SOD activities, T-AOC, and GSH-Px activities in piglets, indicating hepatic lipid peroxidation. This study further suggested that the effect might be mediated via the Pregnane X Receptor (PXR) and Constitutive Androstane Receptor (CAR) pathways [20]. Corroborating this, another investigation reported that feeding post-weaning piglets 1 mg/kg ZEA for 22 days significantly suppressed GSH-Px and SOD activities and elevated MDA content in both serum and liver, confirming a severe compromise of the systemic antioxidant defense system [34]. Notably, even lower doses may perturb oxidative homeostasis. Exposure to 0.316 mg/kg ZEA for 18 days upregulated hepatic GSH-Px gene expression but downregulated splenic SOD gene expression, implying tissue-specific oxidative stress responses [35]. Furthermore, research on intestinal antioxidant capacity revealed that dietary ZEA induces oxidative stress in the jejunum of piglets by activating the Keap1-Nrf2 signaling pathway, evidenced by upregulated expression of its downstream target genes quinone oxidoreductase 1 (NQO1), heme oxygenase 1 (HO1), and modifier subunit of glutamate-cysteine ligase (GCLM) [18]. In the present study, systemic antioxidant capacity was compromised, as reflected by reduced serum T-AOC and GSH-Px activity. Hepatic lipid peroxidation was evident across all treatment groups, accompanied by diminished antioxidant enzyme activities. In the intestine, oxidative stress was most pronounced in the jejunum and ileum, with the lowest ZEA dose showing minimal effects. These findings suggest that oxidative stress may be an important mechanism of ZEA toxicity. This notion is further supported by our Spearman correlation analysis, which revealed significant associations between antioxidant markers and multi-organ parameters. Future investigations should focus on elucidating the precise molecular patterns and mechanisms through detailed analyses of gene and protein expression in these target tissues.

In the context of this oxidative stress, the impact of ZEA on the growth performance of weaned piglets appears to be influenced by the exposure dose, duration, and the form of contamination. A study reported that dietary purified ZEA at 290 ppb (0.29 mg/kg) for 22 days did not affect ADG or ADFI in weaned piglets [36]. Marin et al. [35] reported no significant alterations in growth parameters after feeding piglets a diet supplemented with 0.316 mg/kg of purified ZEA for 18 days. In contrast, Song et al. [37] observed a significant reduction in final BW following 28-day exposure to 0.4 mg/kg ZEA from naturally contaminated feed, though the FI and F:G parameters were unaffected. In our study, a significant increase in the F:G ratio was observed only at the highest dose and in the later phase, while final BW showed a non-significant downward trend across all treatment groups. This pattern suggests that purified ZEA at doses up to 0.6 mg/kg may induce only marginal growth suppression in piglets. The more pronounced effects reported in some studies using naturally contaminated materials could be attributable to the synergistic interactions of ZEA with other co-occurring mycotoxins. To clearly define the specific effect of ZEA on piglet growth performance and to assess the potential risk of long-term, low-level exposure, further studies with larger sample sizes and extended experimental durations are warranted.

In addition to its effects on growth, ZEA exposure also influenced hematological parameters, which are crucial for systemic homeostasis. Research indicates that ZEA may exert certain toxic effects on hematological parameters of piglets, though findings across studies are inconsistent. A study employing daily oral gavage reported that administration of 5–15 µg/kg BW ZEA to pre-pubertal gilts significantly decreased hematological parameters, including WBC, RBC, HGB, HCT, and MPV [38]. In contrast, dietary exposure to 0.8 mg/kg ZEA for four weeks did not alter any hematological indices in piglets [39]. Furthermore, Jakimiuk et al. [40] reported that feeding weaned piglets a diet containing 0.1 mg/kg ZEA for 42 days caused no significant changes in erythrocyte-related indices (RBC, HGB, HCT, MCH, etc.) or WBC, although a significant increase in the counts of neutrophilic and acidophilic granulocytes was observed. In this study, hematological parameters remained largely unchanged during the early and middle phases. However, later-phase alterations were observed, including reduced erythrocyte-related indices and changes in platelet parameters. The reductions in HGB and HCT levels suggest that ZEA may interfere with erythropoiesis or induce erythrocyte damage. Oxidative stress is known to promote erythrocyte membrane lipid peroxidation and hemolysis, which could account for the decreased red blood cell indices observed in this study [41]. Consistent with this, our Spearman correlation analysis showed that antioxidant markers (T-AOC, SOD, GSH-Px) were positively correlated with blood HGB and HCT, whereas MDA was negatively correlated with these parameters, further linking oxidative stress to erythrocyte impairment.

Evaluation of serum biochemistry provided further insight into ZEA’s systemic effects. According to prior reports, the effects of high-dose ZEA on the serum biochemistry of piglets primarily manifest as alterations in kidney-related parameters. Studies have shown that dietary exposure to 1 mg/kg ZEA significantly increased serum levels of UN, CREA, and TBIL in piglets, indicating potential renal injury [42]. Another study using the same dose further confirmed the nephrotoxic effects of ZEA, as evidenced by elevated serum urea and CREA levels in treated piglets [34]. In contrast, research on low-dose ZEA (0.1 mg/kg) indicated no significant effects on serum TP, TBIL, AST, or ALT in piglets, with only a transient increase in ALB observed during the early phase [40]. In the present study, serum biochemistry was largely unaffected during the early phase, with only sporadic and generally mild alterations observed in some indices. A transient elevation in AST was noted in the middle and later phases. Whether these changes represent true toxic effects requires comprehensive evaluation alongside other physiological and histopathological endpoints.

Given the sensitivity of immune cells to redox imbalance, the observed oxidative stress may also influence immune function [43]. The present study observed an initial increase in serum immunoglobulins following ZEA exposure. Specifically, the ZEA0.3 group exhibited significantly elevated levels of serum IgA, IgG, and IgM on day 14. However, these differences disappeared by the later stages of the trial, with no significant effects observed across treatment groups. This temporal pattern indicates a transient immunostimulatory effect. This finding aligns with the study by Su et al. [42], which reported that feeding piglets 1 mg/kg ZEA for 28 days significantly increased serum IgA, IgG, and IgM levels, highlighting ZEA’s immunostimulatory potential. Conversely, another study reported that a lower dose of 0.8 mg/kg ZEA led to significant reductions in serum IgG and IgM [39]. Furthermore, Yang et al. [44] found that exposure to dietary ZEA at 1.1–3.2 mg/kg for 18 days caused no significant change in serum IgG at 1.1 mg/kg, but significantly decreased it at the higher doses of 2.0 and 3.2 mg/kg. Taken together, these findings reveal a complex, dose- and time-dependent modulation of the immunoglobulin system by ZEA in piglets.

Immune cytokine profiles were also altered by ZEA exposure, indicative of modulated inflammatory activity. A study shows that 0.316 mg/kg ZEA upregulates the levels of hepatic pro-inflammatory cytokines, including TNF-α, IL-1β, IL-6, IL-8 and IFN-γ, while downregulating the levels of anti-inflammatory cytokines, such as IL-4 and IL-10, in piglets [45]. At higher doses (2.0–3.2 mg/kg), ZEA reduced splenic IL-2 levels and upregulated the expression of pro-inflammatory cytokine genes IL-1β and IL-6 in piglets, indicating splenic immunotoxicity [46]. In vitro studies demonstrated that ZEA upregulates the expression of IL-1β, IL-6, and IL-2 at both transcriptional and protein levels in porcine intestinal epithelial cells (IPEC-J2), thereby inducing a marked inflammatory response. Further investigation suggested that ZEA-induced oxidative stress may serve as a core initiating mechanism underlying this immunotoxic process [47]. Furthermore, the immunotoxicity of ZEA may extend transgenerationally, impairing immune function in offspring [48]. Early in the trial, serum cytokine profiles were markedly altered, characterized by elevated pro-inflammatory cytokines and suppressed anti-inflammatory cytokines. These changes attenuated over time, though suppression of anti-inflammatory cytokines persisted. Similarly, anti-inflammatory cytokines were suppressed in hepatic and intestinal tissues. These results suggest that even ZEA at or below regulatory levels can durably alter the immune status of piglets. Notably, while these are co-occurring observations, the biologically plausible link between oxidative stress and inflammation, coupled with their co-localization and temporal synchrony in our study, is consistent with the hypothesis that the oxidative stress occurring in the serum, liver, and intestine may contribute to the observed persistent immunodysregulation. This hypothesis is further supported by our Spearman correlation analysis. It demonstrated that antioxidant markers (T-AOC, SOD, GSH-Px) were positively correlated with IL-4 and IL-10, but negatively correlated with TNF-α and IL-1β, across multiple tissues. In contrast, MDA content was positively correlated with TNF-α and IL-1β, but negatively correlated with IL-4 and IL-10. Future studies should directly test this causal relationship.

The integrity of intestinal morphology is a prerequisite for normal physiological functions in animals. VH, CD, and VH/CD ratio are key indicators for assessing intestinal digestion and absorption capacity. During feed ingestion, the intestine comes into direct contact with and participates in the absorption and metabolism of ZEA, which can consequently induce intestinal damage and compromise both absorptive and barrier functions [49,50]. Previous research demonstrated that a 42-day exposure to 2 mg/kg ZEA significantly reduced ileal VH, increased CD, and lowered the VH/CD ratio in piglets, indicating severe ileal structural damage and altered crypt-villus axis function [51]. Another study reported that oral administration of ZEA at 40 µg/kg BW for 6 weeks led to numerically reduced jejunal VH and VH/CD in piglets, although these changes were not statistically significant [52]. In this study, ileal VH was reduced across all treatment groups, with a corresponding decrease in the VH/CD ratio at lower doses. The ileal morphological alterations observed occurred in the context of generalized oxidative stress. This link is corroborated by further Spearman correlation analysis, which showed positive correlations between serum GSH-Px and ileal VH and VH/CD ratio, and negative correlations between liver MDA and the same two parameters, suggesting that systemic and hepatic redox status is closely linked to intestinal morphological integrity. While a direct causal link in the ileum cannot be established here, the overall pro-oxidant state induced by ZEA likely contributes to a microenvironment that exacerbates epithelial injury and hampers repair across intestinal segments. Histopathological lesions across multiple intestinal segments corroborated the morphometric findings. Despite structural changes, serum markers of intestinal permeability were largely unaffected, with only a transient elevation in LPS. This apparent discrepancy between structural damage and permeability markers may reflect the complexity of intestinal barrier function, where structural damage does not always translate into immediate changes in systemic markers, possibly due to compensatory mechanisms. Taken together, these results suggest that while ZEA at doses below 0.6 mg/kg induces structural alterations in multiple intestinal segments, these changes may be insufficient to cause persistent, systemically detectable barrier dysfunction under the conditions of this study.

Beyond the intestine, ZEA also exhibited low-grade toxicity in other vital organs, with the liver showing particular sensitivity. Although dietary ZEA exposure at 0.075–0.6 mg/kg did not significantly affect the heart, liver, spleen, lungs, or kidney indices, histopathological examination revealed tissue-specific morphological alterations. Mild hepatic lesions were detectable even at the lowest ZEA dose, indicating high hepatic sensitivity. The observed hyperplastic changes, together with the elevated hepatic MDA content in all ZEA-treated groups, suggest that oxidative stress may contribute to these cellular alterations, reinforcing its role as a key contributor to early hepatic injury at low ZEA exposure levels. Renal lesions were observed only at 0.15 and 0.6 mg/kg. Collectively, the data indicate that low-dose ZEA induces subtle yet consistent histopathological alterations in the liver and kidney, which may be associated with concurrent oxidative damage. This underscores the potential of combining oxidative stress parameters with tissue histology as more sensitive biomarkers for detecting early toxicity than relying on traditional organ indices alone.

Considering the systemic oxidative stress observed in this study, we also examined whether ZEA exerts endocrine-disrupting effects. Reproductive hormone profiles were disrupted across all doses, with suppression of FSH, LH, and PRL, and elevation of GnRH. This pattern is consistent with previous reports where higher ZEA doses (1.0 and 1.13 mg/kg) significantly suppressed serum levels of FSH, LH, E_2_, and PROG in piglets [42,53]. It has been established that ZEA exposure can significantly suppress key reproductive hormones, an effect mediated by interference with pituitary signaling and disruption of the hypothalamic-pituitary-gonadal (HPG) axis feedback regulation, ultimately leading to reduced gonadotropin secretion [2,54]. Consistently, a recent review has further confirmed that ZEA disrupts the balance of reproductive hormones and interferes with the secretion of gonadotropins such as FSH and LH [55]. Therefore, our findings indicate that even at doses as low as 0.075 mg/kg, ZEA can disrupt the endocrine system and disturb the balance of reproductive hormone levels in piglets. Histopathological changes in the uterus and ovaries at higher doses further supported the estrogenic disruption. Oxidative stress is known to impair steroidogenesis and disrupt cellular signaling within the HPG axis [56,57]. Consistent with this, our correlation analysis revealed that serum GSH-Px was positively associated with PRL and negatively with GnRH, while intestinal MDA was negatively associated with LH. These correlative findings suggest that systemic antioxidant status and tissue oxidative damage are closely associated with the observed reproductive hormone disruptions. Future research should investigate whether antioxidant intervention can mitigate these reproductive disturbances, which would further clarify the role of redox imbalance in ZEA-mediated reproductive toxicity.

Nevertheless, several limitations of the present study should be acknowledged. As a preliminary study, the relatively small sample size (*n* = 4 per group) may limit the statistical power and increase the susceptibility of the findings to individual variation, potentially affecting the robustness of the conclusions. Our exploratory Spearman correlation analysis revealed significant associations between oxidative stress markers and multi-organ parameters. However, these correlative findings should be validated in larger samples. Therefore, the results should be interpreted with caution. Despite these limitations, the present findings provide preliminary evidence that ZEA induces systemic oxidative stress and subclinical multi-organ toxicity, providing a valid rationale for exploring antioxidant-based mitigation strategies. The integration of dietary antioxidants represents a promising, practical approach to counteract mycotoxin-induced damage in livestock production. Notably, DL-selenomethionine has been shown to alleviate zearalenone-induced oxidative stress in porcine intestinal epithelial cells via the activation of the Nrf2/Keap1 signaling pathway [58]. Additionally, vitamin C has been demonstrated to protect piglet livers against ZEA-induced oxidative stress by modulating nuclear receptors PXR and CAR and their target genes [26], and to ameliorate ZEA-induced reproductive, immunological, and hematological toxicity in weaned piglets [42]. Similarly, lycopene, a potent carotenoid, has been shown to mitigate ZEA-induced oxidative damage and apoptosis in piglet Sertoli cells by activating the Nrf2/HO-1 signaling pathway, enhancing antioxidant enzyme activity, and reducing autophagy [59], as well as to alleviate ZEA-induced renal toxicity in mice by inhibiting oxidative stress, apoptosis, and NLRP3 inflammasome activation [60]. Moreover, a recent study revealed that curcumin inhibits ZEA-induced ferroptosis in porcine intestinal epithelial cells via the p53/SLC7A11/GPX4 pathway, further expanding the understanding of ZEA toxicity mechanisms and the protective role of natural antioxidants [61]. Other natural antioxidants, such as resveratrol and quercetin, have also demonstrated protective effects against ZEA-induced damage in vivo and in vitro, respectively. Resveratrol alleviated intestinal barrier dysfunction in mice through the NF-κB/Nrf2/HO-1 pathway [62], while quercetin protected porcine renal epithelial cells from apoptosis and necroptosis by inhibiting the CaSR/CaMKII axis [63].

Given these findings and the limitations noted above, future research should focus on validating the present observations with larger cohorts and incorporating more specific oxidative stress markers, such as the reduced glutathione/oxidized glutathione (GSH/GSSG) ratio and 8-hydroxy-2’-deoxyguanosine (8-OHdG), to further characterize the role of redox imbalance, as well as conducting applied studies to evaluate the efficacy of novel antioxidants against realistic chronic low-dose ZEA exposure in piglets, while also proactively exploring novel natural antioxidants. Key objectives should encompass determining the optimal dosage and form of antioxidant delivery, elucidating their precise molecular mechanisms of protection, and assessing the economic feasibility of their inclusion in commercial feed. Ultimately, effective nutritional interventions incorporating antioxidants represent an essential strategy to safeguard animal health, welfare, and productivity in the face of unavoidable ZEA contamination.

## 5. Conclusions

In conclusion, this preliminary study suggests that low doses of purified ZEA may induce systemic oxidative stress as a pivotal and sensitive response in weaned female piglets. This redox imbalance, characterized by compromised antioxidant capacity and elevated lipid peroxidation, was accompanied by a spectrum of subclinical multi-organ toxicity. These included inflammatory dysregulation, ileal morphological impairment, hepatic histopathological damage, and reproductive hormone disruption. Notably, decreased IL-4 levels in serum, liver, and intestine, reduced ileal VH, mild hepatic histopathological lesions, and suppressed serum LH level were observed even at the lowest dose of 0.075 mg/kg. Moreover, Spearman correlation analysis revealed that oxidative stress markers were significantly correlated with these multi-organ toxicity parameters. Our findings suggest that oxidative stress is not merely a concomitant effect, but a plausible central mechanism contributing to the observed multi-faceted toxicity at low exposure levels. Therefore, the monitoring of oxidative stress parameters constitutes a highly sensitive strategy for the early assessment of ZEA-induced health risks. This preliminary study highlights the need to integrate redox status biomarkers into future risk assessment strategies and provides a strong rationale for the development and implementation of targeted antioxidant interventions to mitigate the toxicity of ZEA in livestock production. However, it should be noted that the small sample size (*n* = 4 per group) limits the generalizability of these findings, and future studies with larger sample sizes are warranted to validate the observed effects.

## Figures and Tables

**Table 1:** Effects of ZEA exposure on growth performance in piglets.

Items	Treatments					*p*-Values
	CON	ZEA0.075	ZEA0.15	ZEA0.3	ZEA0.6	
Initial BW (kg)	7.08 ± 0.83	7.13 ± 0.64	7.18 ± 0.93	7.10 ± 0.97	7.10 ± 0.57	1.000
14 d BW (kg)	10.99 ± 1.63	10.78 ± 1.56	10.80 ± 1.66	9.85 ± 0.34	11.60 ± 1.57	0.571
28 d BW (kg)	19.43 ± 3.37	17.40 ± 1.96	17.73 ± 1.82	16.49 ± 1.79	19.11 ± 2.06	0.371
42 d BW (kg)	29.93 ± 4.82	27.71 ± 2.36	28.04 ± 3.62	27.24 ± 1.86	28.50 ± 1.93	0.784
0–14 d						
ADG (kg)	0.28 ± 0.08	0.26 ± 0.08	0.26 ± 0.07	0.20 ± 0.07	0.32 ± 0.07	0.261
ADFI (kg)	0.48 ± 0.09	0.46 ± 0.10	0.44 ± 0.10	0.35 ± 0.11	0.53 ± 0.07	0.180
F:G	1.76 ± 0.23	1.80 ± 0.26	1.71 ± 0.11	1.82 ± 0.31	1.68 ± 0.29	0.923
15–28 d						
ADG (kg)	0.60 ± 0.13	0.47 ± 0.12	0.49 ± 0.02	0.47 ± 0.11	0.54 ± 0.04	0.287
ADFI (kg)	0.98 ± 0.20	0.80 ± 0.16	0.86 ± 0.09	0.84 ± 0.11	0.95 ± 0.06	0.321
F:G	1.64 ± 0.11	1.72 ± 0.19	1.74 ± 0.12	1.80 ± 0.20	1.77 ± 0.05	0.586
29–42 d						
ADG (kg)	0.75 ± 0.11	0.74 ± 0.03	0.74 ± 0.15	0.77 ± 0.05	0.67 ± 0.03	0.629
ADFI (kg)	1.40 ± 0.25	1.27 ± 0.04	1.36 ± 0.23	1.34 ± 0.05	1.38 ± 0.11	0.815
F:G	1.87 ± 0.11 ^B^	1.73 ± 0.02 ^B^	1.86 ± 0.10 ^B^	1.75 ± 0.06 ^B^	2.06 ± 0.20 ^A^	0.007
0–42 d						
ADG (kg)	0.54 ± 0.10	0.49 ± 0.06	0.50 ± 0.08	0.48 ± 0.03	0.51 ± 0.03	0.673
ADFI (kg)	0.96 ± 0.17	0.84 ± 0.07	0.89 ± 0.13	0.84 ± 0.05	0.95 ± 0.07	0.420
F:G	1.76 ± 0.11	1.73 ± 0.10	1.79 ± 0.04	1.76 ± 0.06	1.87 ± 0.06	0.182

Note: BW, body weight; ADG, average daily gain; ADFI, average daily feed intake; F:G, feed-to-gain ratio. Data are presented as mean ± SD, *n* = 4. ^A,B^, different letters mean a statistical difference (*p* < 0.01).

**Table 2:** Effects of ZEA exposure on major organ indices in piglets (g/kg).

Items	Treatments					*p*-Values
	CON	ZEA0.075	ZEA0.15	ZEA0.3	ZEA0.6	
Heart index	5.05 ± 0.83	5.17 ± 0.44	4.54 ± 0.37	5.15 ± 0.95	4.80 ± 0.59	0.647
Liver index	25.58 ± 1.07	23.80 ± 2.39	26.86 ± 4.28	24.05 ± 2.88	24.13 ± 4.42	0.639
Spleen index	1.78 ± 0.30	1.71 ± 0.27	1.83 ± 0.25	2.03 ± 0.30	1.79 ± 0.32	0.622
Lung index	8.81 ± 0.85	9.91 ± 1.43	9.92 ± 1.90	11.45 ± 1.42	9.19 ± 1.36	0.148
Kidney index	4.22 ± 0.30	4.65 ± 0.24	4.98 ± 0.94	4.50 ± 0.62	4.15 ± 0.82	0.403

Note: Data are presented as mean ± SD, *n* = 4.

**Table 3:** Effects of ZEA exposure on hematological parameters in piglets at day 42.

Items	Treatments					*p*-Values
	CON	ZEA0.075	ZEA0.15	ZEA0.3	ZEA0.6	
42 d						
WBC (10^9^/L)	17.35 ± 8.82	18.32 ± 6.55	17.13 ± 4.97	12.23 ± 1.60	13.58 ± 1.05	0.466
RBC (10^12^/L)	5.96 ± 0.59	5.51 ± 0.53	5.02 ± 0.54	5.00 ± 0.55	5.48 ± 0.37	0.105
HGB (g/L)	101.25 ± 8.22 ^A^	92.00 ± 5.29 ^B^	84.00 ± 2.45 ^B^	87.75 ± 3.50 ^B^	88.75 ± 3.86 ^B^	0.003
HCT (%)	31.95 ± 2.85 ^a^	29.48 ± 2.44 ^ab^	26.85 ± 0.97 ^b^	28.15 ± 0.54 ^b^	27.88 ± 1.02 ^b^	0.012
MCV (fL)	53.68 ± 1.30	53.53 ± 2.24	53.80 ± 4.23	56.68 ± 5.27	51.00 ± 2.23	0.278
MCH (pg)	17.03 ± 0.36	16.78 ± 1.14	16.85 ± 1.39	17.63 ± 1.21	16.23 ± 0.67	0.456
MCHC (g/L)	317.00 ± 8.98	312.50 ± 11.47	312.75 ± 2.99	311.50 ± 9.15	318.25 ± 4.72	0.699
RDW-SD (fL)	37.08 ± 1.84	39.95 ± 2.06	38.23 ± 3.81	38.68 ± 2.22	34.80 ± 1.99	0.093
RDW-CV (%)	21.43 ± 0.73	22.68 ± 1.37	21.50 ± 3.37	20.53 ± 1.85	21.10 ± 0.32	0.593
PLT (10^9^/L)	288.25 ± 65.61	313.50 ± 57.74	381.25 ± 56.96	258.50 ± 65.20	279.50 ± 53.87	0.088
PDW (fL)	16.28 ± 0.79 ^AB^	13.20 ± 1.42 ^C^	17.45 ± 1.26 ^A^	14.63 ± 2.17 ^BC^	13.55 ± 1.38 ^C^	0.004
MPV (fL)	11.83 ± 1.09 ^AB^	10.25 ± 0.10 ^C^	12.50 ± 0.70 ^A^	11.13 ± 0.93 ^BC^	10.78 ± 0.39 ^BC^	0.005

Note: WBC, white blood cell count; RBC, red blood cell count; HGB, hemoglobin; HCT, hematocrit; MCV, mean corpuscular volume; MCH, mean corpuscular hemoglobin; MCHC, mean corpuscular hemoglobin concentration; RDW-SD, standard deviation of red cell distribution width; RDW-CV, coefficient of variation in red cell distribution width; PLT, platelet count; PDW, platelet distribution width; MPV, mean platelet volume. Data are presented as mean ± SD, *n* = 4. ^A–C^, different letters mean a statistical difference (*p* < 0.01). ^a,b^, different letters mean a statistical difference (*p* < 0.05).

**Table 4:** Effects of ZEA exposure on serum biochemical parameters in piglets at day 42.

Items	Treatments					*p*-Values
	CON	ZEA0.075	ZEA0.15	ZEA0.3	ZEA0.6	
42 d						
TP (g/L)	60.10 ± 4.90	58.04 ± 3.93	57.50 ± 3.51	57.93 ± 4.52	57.24 ± 2.00	0.849
ALB (g/L)	28.81 ± 1.51	24.63 ± 3.64	24.57 ± 2.14	26.78 ± 2.44	24.14 ± 1.71	0.071
GLB (g/L)	31.30 ± 4.30	33.41 ± 5.54	32.93 ± 5.16	31.14 ± 4.64	33.09 ± 1.85	0.919
CREA (µmol/L)	97.39 ± 10.97	83.53 ± 10.82	83.28 ± 13.49	85.14 ± 15.54	90.79 ± 6.91	0.422
UN (mg/dL)	11.38 ± 1.09	9.68 ± 1.13	10.03 ± 0.98	10.70 ± 2.53	10.99 ± 0.70	0.472
GLU (mmol/L)	4.93 ± 0.46	4.48 ± 2.09	4.80 ± 0.82	5.01 ± 1.08	4.95 ± 0.74	0.970
AST (U/L)	42.41 ± 6.66 ^b^	77.78 ± 18.48 ^a^	60.32 ± 4.88 ^ab^	53.65 ± 13.84 ^b^	51.91 ± 11.31 ^b^	0.011
ALT (U/L)	45.22 ± 3.68	59.63 ± 10.64	59.17 ± 12.84	59.86 ± 5.21	62.66 ± 6.56	0.076
TBIL (µmol/L)	5.74 ± 2.88	6.20 ± 1.96	5.70 ± 0.92	6.00 ± 1.77	4.89 ± 1.03	0.879
ALP (U/L)	207.31 ± 28.16	221.58 ± 61.42	232.85 ± 90.07	220.20 ± 73.93	193.44 ± 25.95	0.910
CHE (U/L)	137.64 ± 91.16	143.08 ± 91.22	198.75 ± 135.64	243.55 ± 76.87	196.05 ± 101.00	0.570
LDH (U/L)	503.10 ± 86.30	532.13 ± 101.74	701.80 ± 83.56	465.09 ± 208.75	536.70 ± 118.21	0.146

Note: TP, total protein; ALB, albumin; GLB, globulin; CREA, creatinine; UN, urea nitrogen; GLU, glucose; AST, aspartate aminotransferase; ALT, alanine aminotransferase; TBIL, total bilirubin; ALP, alkaline phosphatase; CHE, cholinesterase; LDH, lactate dehydrogenase. Data are presented as mean ± SD, *n* = 4. ^a,b^, different letters mean a statistical difference (*p* < 0.05).

**Table 5:** Effects of ZEA exposure on serum antioxidant parameters in piglets.

Items	Treatments					*p*-Values
	CON	ZEA0.075	ZEA0.15	ZEA0.3	ZEA0.6	
14 d						
GSH-Px (U/mL)	162.56 ± 2.81 ^A^	158.47 ± 1.86 ^B^	162.66 ± 2.82 ^A^	166.14 ± 1.96 ^A^	165.65 ± 1.60 ^A^	0.002
SOD (U/mL)	83.30 ± 10.04	84.07 ± 1.93	71.97 ± 2.39	81.60 ± 5.66	80.94 ± 3.65	0.051
T-AOC (U/mL)	9.83 ± 1.23 ^A^	7.84 ± 0.38 ^BC^	7.20 ± 0.47 ^C^	8.60 ± 0.36 ^B^	7.65 ± 0.62 ^BC^	0.001
MDA (nmol/mL)	3.22 ± 0.12	3.09 ± 0.64	3.23 ± 0.59	3.10 ± 0.16	3.21 ± 0.16	0.974
28 d						
GSH-Px (U/mL)	163.80 ± 1.43	161.63 ± 4.44	154.37 ± 13.22	162.65 ± 1.85	161.23 ± 3.58	0.320
SOD (U/mL)	98.67 ± 4.86 ^A^	92.27 ± 5.01 ^AB^	82.03 ± 4.82 ^C^	84.87 ± 5.94 ^BC^	89.83 ± 4.48 ^BC^	0.003
T-AOC (U/mL)	10.78 ± 1.03 ^A^	9.26 ± 0.56 ^B^	8.33 ± 0.33 ^B^	8.63 ± 0.20 ^B^	8.81 ± 0.93 ^B^	0.001
MDA (nmol/mL)	2.88 ± 0.71	2.93 ± 0.82	3.57 ± 0.40	3.22 ± 0.46	2.90 ± 0.53	0.462
42 d						
GSH-Px (U/mL)	172.43 ± 4.04 ^A^	159.77 ± 6.56 ^B^	163.74 ± 4.27 ^B^	165.65 ± 1.03 ^B^	164.87 ± 1.69 ^B^	0.008
SOD (U/mL)	90.59 ± 16.38	89.87 ± 7.63	80.33 ± 5.25	97.00 ± 3.65	98.57 ± 9.53	0.110
T-AOC (U/mL)	10.83 ± 0.46	8.62 ± 1.93	8.41 ± 0.32	9.64 ± 1.49	10.32 ± 1.25	0.063
MDA (nmol/mL)	3.22 ± 0.34 ^AB^	3.32 ± 0.26 ^A^	3.57 ± 0.44 ^A^	2.76 ± 0.10 ^B^	2.73 ± 0.37 ^B^	0.009

Note: GSH-Px, glutathione peroxidase; SOD, superoxide dismutase; T-AOC, total antioxidant capacity; MDA, malondialdehyde. Data are presented as mean ± SD, *n* = 4. ^A–C^, different letters mean a statistical difference (*p* < 0.01).

**Table 6:** Effects of ZEA exposure on serum immunoglobulin levels in piglets (g/L).

Items	Treatments					*p*-Values
	CON	ZEA0.075	ZEA0.15	ZEA0.3	ZEA0.6	
14 d						
IgA	1.09 ± 0.05 ^B^	1.35 ± 0.19 ^B^	1.21 ± 0.26 ^B^	1.97 ± 0.30 ^A^	1.21 ± 0.07 ^B^	<0.001
IgG	15.85 ± 1.15 ^C^	19.01 ± 1.18 ^B^	16.25 ± 2.84 ^C^	22.98 ± 0.37 ^A^	16.40 ± 1.21 ^C^	<0.001
IgM	1.83 ± 0.05 ^B^	2.28 ± 0.29 ^B^	2.10 ± 0.58 ^B^	3.64 ± 0.80 ^A^	2.22 ± 0.18 ^B^	0.001
28 d						
IgA	1.33 ± 0.03	1.53 ± 0.49	1.39 ± 0.10	1.50 ± 0.48	1.50 ± 0.07	0.864
IgG	18.55 ± 0.94	20.67 ± 2.01	19.24 ± 4.87	19.85 ± 2.95	22.12 ± 0.17	0.430
IgM	2.35 ± 0.08 ^B^	2.50 ± 0.14 ^B^	2.42 ± 0.14 ^B^	2.46 ± 0.35 ^B^	2.98 ± 0.16 ^A^	0.003
42 d						
IgA	1.46 ± 0.17	1.50 ± 0.17	1.49 ± 0.10	1.58 ± 0.12	1.61 ± 0.10	0.509
IgG	19.55 ± 2.20	20.86 ± 2.02	20.08 ± 1.28	19.80 ± 1.70	21.61 ± 1.88	0.518
IgM	2.54 ± 0.17	3.03 ± 0.29	2.92 ± 0.09	2.65 ± 0.18	3.02 ± 0.53	0.103

Note: IgA, immunoglobulin A; IgG, immunoglobulin G; IgM, immunoglobulin M. Data are presented as mean ± SD, *n* = 4. ^A–C^, different letters mean a statistical difference (*p* < 0.01).

## Data Availability

The original contributions presented in this study are included in the article/Supplementary Materials. Further inquiries can be directed to the corresponding author.

## Supplementary Materials

The following supporting information can be downloaded at https://www.mdpi.com/article/10.3390/antiox15040496/s1: Table S1: The determination of mycotoxins in treatment diets (µg/kg); Table S2: Effects of ZEA exposure on hematological parameters in piglets at days 14 and 28; Table S3: Effects of ZEA exposure on serum biochemical parameters in piglets at days 14 and 28; Table S4: Effects of ZEA exposure on antioxidant parameters in the liver and intestine of piglets; Table S5: Effects of ZEA exposure on immune cytokine levels in the liver and intestine of piglets (pg/mg prot). Figure S1: Effects of ZEA exposure on liver immunoglobulin levels in piglets.
