# Supplementary material for: A Preliminary Study on the Effects of Low Doses of Purified Zearalenone in Weaned Female Piglets: A Multi-Organ Toxicity Investigation
Source: Antioxidants (Basel). 2026 Apr 16;15(4):496. doi: 10.3390/antiox15040496 (PMC13113162; doi:10.3390/antiox15040496)
Supplement: Supplementary file 1 [file antioxidants-15-00496-s001.zip › antioxidants-4162912-supplementary.pdf]

Supplementary Materials: Purified Zearalenone Induces Systemic Oxidative Stress and Subclinical Multi-Organ Toxicity in Weaned Piglets below the Chinese Regulatory Limit

Ying Liu [1, 2], Qiaomin Duan [1, 2], Ruiqi Tan [1, 2], Sunlin Luo [1, 2], Wenjun He [1, 2], Wenjun Yang [1, 2], and Yiqiang Chen [1, 2, *]

1 State Key Laboratory of Animal Nutrition and Feeding, College of Animal Science and Technology, China Agricultural University, Beijing 100193, China

2 Ministry of Agriculture and Rural Affairs Key Laboratory of Feed Safety and Biological Efficacy, China Agricultural University, Beijing 100193, China

* Correspondence: yqchen@cau.edu.cn (Y.C.)

Table S1. The determination of mycotoxins in treatment diets (μg/kg).

Treatments	Addition	Measured values of mycotoxins					
		ZEA	DON	AFB_1	OTA	T-2	FB_1
d 0–14							
CON	0	2.69	45.73	1.15			
ZEA0.075	75	75.75	18.80	0.66			
ZEA0.15	150	124.36	19.08	1.22		< LOQ	
ZEA0.3	300	282.29	24.59	0.90			
ZEA0.6	600	660.44	35.64	0.65			
d 15–42							
CON	0	8.31	25.48	0.81			
ZEA0.075	75	69.63	36.27	0.71			
ZEA0.15	150	155.64	18.32	0.87		< LOQ	
ZEA0.3	300	334.65	29.86	0.73			
ZEA0.6	600	562.18	24.97	0.79			

Note: < LOQ, lower than the limit of quantification. The LOQ for OTA, T-2, and FB_1 were 0.5, 0.5, and 5 μg/kg, respectively.

Table S2. Effects of ZEA exposure on hematological parameters in piglets at days 14 and 28.

Items	Treatments					SEM	*p*-Values
	CON	ZEA0.075	ZEA0.15	ZEA0.3	ZEA0.6		
14 d							
WBC (10^9/L)	14.91	15.36	13.14	15.17	13.72	1.81	0.880
RBC (10^{12}/L)	5.98	6.02	5.86	5.78	6.61	0.41	0.643
HGB (g/L)	109.00	106.00	102.25	106.25	119.75	7.09	0.498
HCT (%)	40.58	40.48	36.83	38.73	44.60	2.23	0.210
MCV (fL)	68.23	67.55	62.88	67.08	68.13	2.36	0.493
MCH (pg)	18.28	17.68	17.40	18.40	18.23	0.50	0.570
MCHC (g/L)	268.50	262.00	277.25	274.25	267.75	5.60	0.379
RDW-SD (fL)	50.25	54.53	48.48	46.85	51.13	3.01	0.472
RDW-CV (%)	22.15	24.43	23.55	21.35	23.03	0.98	0.254
PLT (10^9/L)	238.50	383.25	295.25	324.50	287.50	65.79	0.633
PDW (fL)	20.03	20.15	19.30	18.93	20.43	1.11	0.860
MPV (fL)	13.85	13.78	13.48	13.48	13.55	0.26	0.772
28 d							
WBC (10^9/L)	12.91	15.28	15.82	12.18	14.10	1.94	0.651
RBC (10^{12}/L)	5.06	5.14	4.83	4.35	5.44	0.30	0.184
HGB (g/L)	85.75	91.73	79.25	77.00	89.00	6.31	0.443
HCT (%)	27.05	31.60	25.38	24.60	27.65	2.85	0.480
MCV (fL)	53.58	58.08	52.85	56.78	50.98	2.31	0.229
MCH (pg)	16.95	17.50	16.45	17.73	16.40	0.49	0.256
MCHC (g/L)	316.75	295.83	312.25	312.75	321.75	7.55	0.211
RDW-SD (fL)	38.33	40.08	37.63	41.28	37.30	2.09	0.629
RDW-CV (%)	21.63	20.55	21.80	21.75	22.15	0.78	0.666
PLT (10^9/L)	251.25	312.68	410.50	289.50	299.75	43.44	0.170
PDW (fL)	23.78	21.78	20.05	19.10	23.00	1.27	0.099
MPV (fL)	13.63	13.35	12.88	13.18	13.93	0.40	0.423

Note: WBC, white blood cell count; RBC, red blood cell count; HGB, hemoglobin; HCT, hematocrit; MCV, mean corpuscular volume; MCH, mean corpuscular hemoglobin; MCHC, mean corpuscular hemoglobin concentration; RDW-SD, standard deviation of red cell distribution width; RDW-CV, coefficient of variation of red cell distribution width; PLT, platelet count; PDW, platelet distribution width; MPV, mean platelet volume. SEM, standard error of the mean. $n = 4$.

Table S3. Effects of ZEA exposure on serum biochemical parameters in piglets at days 14 and 28.

Items	Treatments					SEM	*p*-Values
	CON	ZEA0.075	ZEA0.15	ZEA0.3	ZEA0.6		
14 d							
TP (g/L)	53.18[b]	54.10[ab]	51.08[b]	61.90[a]	48.99[b]	2.70	0.039
ALB (g/L)	25.45	24.08	24.42	23.23	22.77	1.72	0.825
GLB (g/L)	27.71	30.02	26.66	38.68	26.22	3.62	0.143
CREA (μmol/L)	73.91	74.58	75.26	74.11	76.40	3.69	0.989
UN (mg/dL)	15.48	18.04	13.14	14.17	12.30	2.00	0.328
GLU (mmol/L)	3.62	4.07	4.99	3.90	4.12	0.64	0.642
AST (U/L)	74.44	98.64	66.21	64.75	69.63	13.84	0.435
ALT (U/L)	43.04	57.71	65.90	44.57	56.11	8.40	0.314
TBIL (μmol/L)	7.41	7.88	8.45	8.04	8.16	1.04	0.966
ALP (U/L)	229.40	221.22	235.75	135.06	233.02	32.57	0.198
CHE (U/L)	374.41	281.25	339.54	245.53	339.96	73.69	0.741
LDH (U/L)	649.85	684.49	590.95	810.43	652.79	147.54	0.870
28 d							
TP (g/L)	54.86	55.75	51.87	54.74	53.92	2.89	0.899
ALB (g/L)	25.24	22.03	20.75	22.12	20.92	1.66	0.357
GLB (g/L)	29.62	33.72	31.12	32.62	33.00	4.07	0.954
CREA (μmol/L)	81.94	73.13	75.64	72.91	83.25	5.56	0.556
UN (mg/dL)	13.22	13.37	14.26	9.70	10.70	1.45	0.178
GLU (mmol/L)	3.80	4.14	4.28	4.19	4.21	0.52	0.969
AST (U/L)	42.35[b]	91.56[a]	68.59[ab]	64.74[ab]	87.83[a]	11.26	0.048
ALT (U/L)	47.54	52.96	57.78	58.20	69.43	5.27	0.100
TBIL(μmol/L)	2.92	7.63	6.07	5.58	6.46	1.08	0.077
ALP (U/L)	235.11	182.31	231.14	170.71	206.68	27.13	0.386
CHE (U/L)	220.06	211.02	326.70	211.01	213.32	60.97	0.611
LDH (U/L)	473.85	644.81	552.96	572.23	723.80	87.23	0.358

Note: TP, total protein; ALB, albumin; GLB, globulin; CREA, creatinine; UN, urea nitrogen; GLU, glucose; AST, aspartate aminotransferase; ALT, alanine aminotransferase; TBIL, total bilirubin; ALP, alkaline phosphatase; CHE, cholinesterase; LDH, lactate dehydrogenase. SEM, standard error of the mean. $n = 4$. [a, b], different letters mean a statistical difference ($p < 0.05$).

Table S4. Effects of ZEA exposure on antioxidant parameters in the liver and intestine of piglets.

Items	Treatments					SEM	*p*-Values
	CON	ZEA0.075	ZEA0.15	ZEA0.3	ZEA0.6		
Liver							
GSH-Px (U/mg prot)	18.90^{A}	17.77^{A}	15.13^{B}	17.40^{A}	17.46^{A}	0.57	0.005
SOD (U/mg prot)	7.64^{A}	7.45^{AB}	5.71^{C}	6.85^{B}	7.27^{AB}	0.21	< 0.001
T-AOC (U/mg prot)	1.43	1.41	1.29	1.30	1.39	0.07	0.515
MDA (nmol/mg prot)	1.37^{B}	1.76^{A}	2.02^{A}	1.91^{A}	1.93^{A}	0.09	0.001
Jejunal mucosa							
GSH-Px (U/mg prot)	17.51^{A}	16.81^{A}	8.71^{C}	13.62^{B}	14.00^{B}	0.48	< 0.001
SOD (U/mg prot)	5.93	5.67	5.02	5.33	5.50	0.32	0.361
T-AOC (U/mg prot)	1.46	1.43	1.34	1.43	1.38	0.09	0.882
MDA (nmol/mg prot)	1.88^{C}	2.09^{BC}	2.44^{A}	2.30^{AB}	2.19^{B}	0.08	0.001
Ileal mucosa							
GSH-Px (U/mg prot)	16.36	15.67	13.51	14.91	15.13	0.69	0.098
SOD (U/mg prot)	6.06^{a}	5.57^{a}	4.50^{b}	5.36^{ab}	5.54^{a}	0.32	0.045
T-AOC (U/mg prot)	1.41	1.33	1.13	1.27	1.33	0.07	0.119
MDA (nmol/mg prot)	1.81^{c}	1.94^{bc}	2.42^{a}	2.35^{ab}	2.13^{abc}	0.13	0.021

Note: GSH-Px, glutathione peroxidase; SOD, superoxide dismutase; T-AOC, total antioxidant capacity; MDA, malondialdehyde. SEM, standard error of the mean. $n = 4$. $^{A–C}$, different letters mean a statistical difference ($p < 0.01$). $^{a–c}$, different letters mean a statistical difference ($p < 0.05$).

Table S5. Effects of ZEA exposure on immune cytokine levels in the liver and intestine of piglets (pg/mg prot).

Items	Treatments					SEM	*p*-Values
	CON	ZEA0.075	ZEA0.15	ZEA0.3	ZEA0.6		
Liver							
TNF-α	4.65	5.18	5.74	5.45	4.88	0.33	0.198
IL-1β	2.07^{c}	2.19^{bc}	2.63^{a}	2.43^{ab}	2.25^{bc}	0.10	0.014
IL-2	17.12	18.53	20.71	19.45	18.50	1.13	0.287
IL-4	1.25^{A}	0.96^{B}	0.84^{B}	0.87^{B}	0.91^{B}	0.07	0.007
IL-10	2.89^{A}	2.46^{B}	1.75^{C}	1.97^{C}	2.13^{BC}	0.13	< 0.001
Jejunal mucosa							
TNF-α	4.47	4.89	5.54	5.44	4.94	0.26	0.066
IL-1β	2.04	2.13	2.35	2.11	2.09	0.12	0.470
IL-2	18.58	20.39	22.61	20.82	20.36	0.87	0.068
IL-4	1.55^{A}	1.21^{B}	0.95^{B}	1.08^{B}	1.16^{B}	0.09	0.006
IL-10	2.76^{A}	2.45^{AB}	1.55^{C}	2.23^{B}	2.31^{AB}	0.15	0.001
Ileal mucosa							
TNF-α	4.54^{B}	4.61^{B}	5.42^{A}	4.73^{B}	4.52^{B}	0.15	0.003
IL-1β	2.14^{B}	2.33^{B}	2.99^{A}	2.54^{B}	2.53^{B}	0.12	0.003
IL-2	21.75	23.77	24.74	24.16	23.62	0.87	0.206
IL-4	1.43^{A}	1.14^{B}	0.81^{D}	0.92^{CD}	1.00^{BC}	0.06	< 0.001
IL-10	3.00^{A}	2.63^{AB}	2.06^{C}	2.25^{BC}	2.36^{BC}	0.13	0.001

Note: TNF-α, tumor necrosis factor-α; IL-1β, interleukin-1β; IL-2, interleukin-2; IL-4, interleukin-4; IL-10, interleukin-4. SEM, standard error of the mean. $n = 4$. $^{A–D}$, different letters mean a statistical difference (*p*

< 0.01). [a–c], different letters mean a statistical difference ($p < 0.05$).

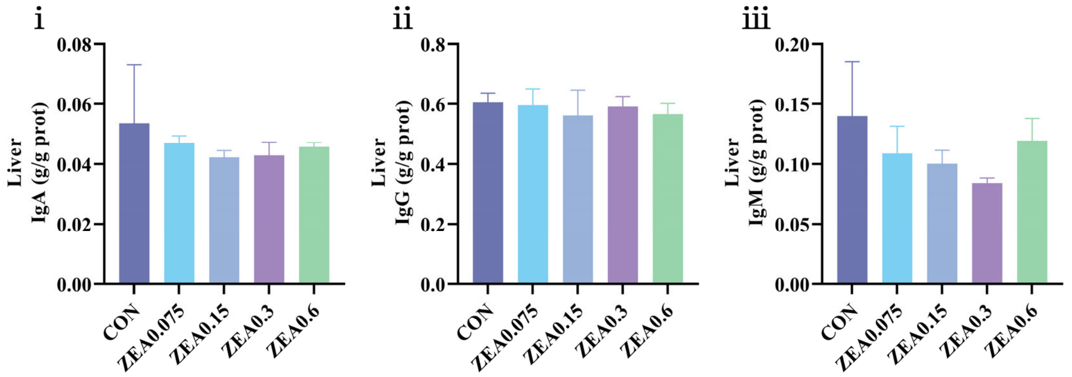

Figure S1. Effects of ZEA exposure on liver immunoglobulin levels in piglets. (i) immunoglobulin A (IgA), (ii) Immunoglobulin G (IgG), (iii) Immunoglobulin M (IgM). Data are presented as means ± SD, n = 4.
